# Efficacy of Commercially Available Invertebrate Predators against *Drosophila suzukii*

**DOI:** 10.3390/insects5040952

**Published:** 2014-11-28

**Authors:** Andrew G. S. Cuthbertson, Lisa F. Blackburn, Neil Audsley

**Affiliations:** The Food and Environment Research Agency, Sand Hutton, York YO41 1LZ, UK; E-Mails: lisa.blackburn@fera.gsi.gov.uk (L.F.B.); neil.audsley@fera.gsi.gov.uk (N.A.)

**Keywords:** *Drosophila suzukii*, natural enemies, predation

## Abstract

*Drosophila suzukii* has been recorded in the UK since the end of 2012. To date, reports of serious damage have been rare. Previous research has demonstrated that there are chemicals available within the UK that are efficient in dealing with *D. suzukii*. However, few effective chemicals for use by the organic sector have been identified; equally the addition of “new” insecticides into previously stable ecosystems can have negative impacts upon natural enemies and so disrupt control strategies that have developed over a period of time. Therefore, there is a need also to screen for potential biological control agents for *D. suzukii*. The following commercially available predatory species were evaluated for their potential to act as control agents for *D. suzukii*: *Orius majusculus*, *Orius laevigatus*, *Atheta coriaria*, *Hypoaspis miles* and *Anthocoris nemoralis*. This set of natural enemies could potentially target several life stages of *D. suzukii* (larvae, pupae and adults). All species, except *H. miles*, fed on *D. suzukii* life stages to some extent. *Hypoaspis miles* displayed no impact upon *D. suzukii* populations. *Anthocoris nemoralis* displayed a tendency to feed upon more male than female adult *D. suzukii* and caused 45% mortality after five days. None of the natural enemies trialed were able to control *D. suzukii* individually. However, these and other non-commercially produced species will all play a role within a given ecosystem in controlling *D. suzukii* populations.

## 1. Introduction

The invasive pest, Spotted Winged Drosophila (SWD), *Drosophila suzukii* (Matsumura) has a wide host range, infesting many varieties of soft fruits [1,2]. Much economic damage has been caused in various regions around the world where *D. suzukii* has become established [2,3,4]. Since the first recording of the pest in the United Kingdom (UK) in 2012 [5], monitoring traps continue to record its presence across the country; though reports of serious damage have been, to date, rare [6]. Several chemical products available within the UK (e.g., Spinosad) have under laboratory conditions shown excellent potential as control agents against SWD [5]. However, organic fruit growers still have limited options as few products appropriate for organic use have been found to be effective against SWD [7]. Equally, the use of chemical insecticides can be very disruptive to natural enemies already being used in integrated pest management strategies within cropping ecosystems [8]. Therefore, there is the need to screen for potential biological control agents for SWD.

Female *D. suzukii* lay their eggs inside the fruit. Here, the larvae grow and consume the fruit. Larvae may then leave the fruit, or remain inside it, to pupate. Following pupation adult flies emerge. Therefore, there are several stages were different natural enemies may be efficient at targeting different lifestages of *D. suzukii*, that is, both on the soil surface as larvae/pupae fall to the ground following leaving the fruit and also in the canopy against adult flies.

Little literature exists on successful biological control of SWD [2]. Several studies have investigated entomopathogenic fungi [5,9]. Here, mortality of SWD ranged widely between species of fungus screened, with no species being determined as offering sufficient control. Direct sprays of fungi onto SWD adults did not kill adult flies quick enough; as a result the next generation of flies began emerging in the feeding media before adult flies that had been treated began to die [5]. These findings demonstrate that pathogen induced mortality alone is not enough to control fly populations. Cuthbertson* et al.* [5] also investigated the use of entomopathogenic nematodes. This study, demonstrated that several species of Steinernematid nematodes did not significantly reduce emerging adult SWD populations from infested blueberries. Predatory bugs from the Genus *Orius* were observed feeding on *D. suzukii* in raspberries, as cited by Walsh *et al.* [2], and *Orius laevigatus* was observed to be present in *D. suzukii* infested fruit trees in Spain [10]. Malagnini *et al*. [11] showed in preliminary studies that *O. majusculus* displayed slight predatory activity against *D. suzukii* whereas *O. laevigatus* offered no predatory activity. Overall, the use of *Orius* spp. for effective control of *D. suzukii* has never been proven.

Studies to determine presence of indigenous parasitoid biological control agents and their efficacy in controlling *D. suzukii* have been undertaken in both North America and Europe by different research groups [12,13]. Under laboratory conditions several naturally occurring parasitoids of drosophilids in France were able to successfully parasitize *D. suzukii*. These included two larval parasitoids, *Leptopilina heterotoma* and *Leptopilina boulardi*, and two pupal parasitoids, *Pachycrepoideus vindemiae* and *Trichopria drosophilae*. Both *Leptopilina* parasitoids displayed high parasitism rates on *D. suzukii*, but because of the strong immune response of the host larvae, they did not give rise to an adult wasp [12]. Therefore, there is a need to determine more efficient invertebrate predators of SWD. To this end, the aim of the current study was to screen the potential of several commercially available invertebrate predators within the UK as control agents against various life stages of SWD.

## 2. Experimental Section

### 2.1. Source of Insects

*Drosophila suzukii* was obtained and cultured as described by Cuthbertson *et al*. [5]. Briefly, flies originated from wild specimens from Northern Italy, collected in the autumn of 2012. These were imported into the UK under a specific license required for importing non-indigenous invertebrates [14]. The flies were held within insect cages at 25 °C, 65% RH and 16:8 h L:D regime and maintained on a mixture of Drosophila diet (Blades Biological, Cowden, UK) and organic blueberries [5].

The predatory species selected for screening were as follows: *Orius majusculus*, *O. laevigatus* (both efficient in thrips control); *Atheta coriaria* (a Staphylinid beetle that is a predator of soil and compost pests); *Hypoaspis miles* (a soil dwelling predatory mite) and *Anthocoris nemoralis* (a generalist predatory bug). These predators were chosen as they had the potential to target multiple *D. suzukii* life stages (larvae, pupae and adults). All control products were supplied by Syngenta Bioline (Little Clacton, UK).

### 2.2. Experimental Arenas

Experimental arenas similar to those described by Tashiro [15] were used to investigate the feeding rates of the individual predatory species on various life stages of *D. suzukii*. The life stages investigated were larvae, pupae and adults. Briefly, the cages consisted of two acrylic plates sandwiching a filter paper covered by a second (1 cm thick) acrylic sheet with a 5 cm diameter aperture drilled in it [15].

Following the method of Cuthbertson *et al.* [16], equal numbers of a given life stage of *D. suzukii* were placed in the arena via the aperture in the middle acrylic plate. Then an individual predatory agent of a given species was added. The aperture was then covered with a filter paper disc and held in place with the upper plate. The whole assembly was secured with rubber bands at each end and maintained in a controlled environment cabinet (25 ± 1 °C, 65% RH, 16:8 h L:D).

### 2.3. The Feeding Rate of Orius majusculus and Orius laevigatus

Experimental arenas were initiated as described above. The potential of *Orius* spp. to feed upon male and female SWD was investigated. Five male SWD were placed inside an arena. Then an individual *O. majusculus* was added. A grain of flower pollen (mixed taxa) was also added as an additional food source for the predatory bug. Controls consisted of equal numbers of male SWD but no predatory bug present. The procedure was replicated seven times and repeated using *O. laevigatus* as the predatory species. The experiment was then also repeated using female SWD as the prey source. Mortality of adult SWD was assessed over the following days.

### 2.4. The Feeding Rate of Atheta coriaria

Experimental arenas were initiated as described above. Ten SWD larvae were placed inside an arena along with commercial Drosophila media [5]. Then an individual *A. coriaria* was introduced. The experiment was replicated 15 times. Mortality was assessed over the following days. Equal number of control arenas were initiated, each containing the same number of larvae but lacking the predator. The procedure was repeated to investigate the impact of *A. coriaria* upon SWD pupae.

### 2.5. The Feeding Rate of Hypoaspis miles

Experimental arenas were initiated as described above. Five SWD larvae were placed inside an arena along with commercial Drosophila media [5]. One predatory mite was then added to the arena. The experiment was replicated 15 times. Mortality of larvae was assessed over the following days. Equal number of control arenas were initiated, each containing the same number of larvae but lacking the predator. The procedure was repeated to investigate the impact of *H. miles* upon SWD pupae.

### 2.6. The Feeding Rate of Anthocoris nemoralis

Again, experimental arenas were initiated as described above. Five SWD adult males were placed inside an arena along with a grain of flower pollen (mixed taxa) as an additional food source. One predatory bug was then added to the arena. The experiment was replicated 15 times. Adult SWD mortality was assessed over the following days. Equal number of control arenas were initiated, each containing the same number of adult flies but lacking the predator. The procedure was repeated to assess the impact of *A. nemoralis* on female SWD.

### 2.7. Data Analysis

Data was statistically analyzed where necessary. Treatments were compared against the control. For analysis the numbers for each life stage in each treatment were combined. Assuming normality and constant variance, Analysis of Variance (ANOVA) was used to test for any significant difference. The threshold for significant difference was *p* < 0.05.

## 3. Results and Discussion

### 3.1. Orius majusculus and Orius laevigatus

**Figure 1:**
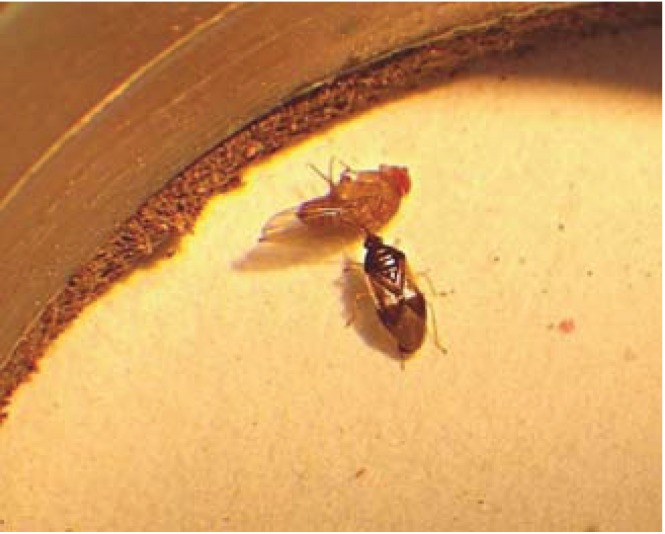
*Orius laevigatus* feeding on an adult *Drosophila suzukii* (photo: Andrew G.S. Cuthbertson^©^).

**Figure 2:**
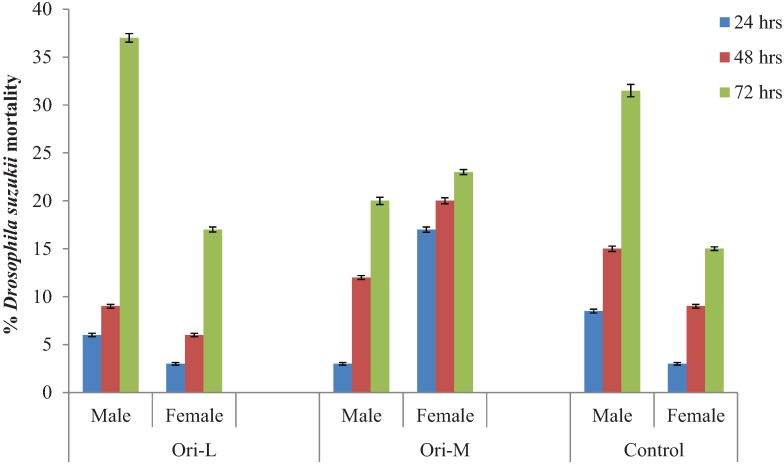
Percentage mortality of *Drosophila suzukii* adults following exposure to *Orius laevigatus* (Ori-L) and *Orius majusculus* (Ori-M).

Within 30 minutes of initiating the experiment an individual *O. laevigatus* was visually observed to feed on an adult female SWD [17] (Figure 1). *Orius laevigatus* fed upon more male than female SWD following 72 h (*p* < 0.001). *Orius laevigatus* significantly reduced male SWD numbers compared to the control (*p* < 0.05) following 72 h (Figure 2). Significantly more mortality of female SWD was caused by *O. laevigatus* compared to controls after 72 h (*p* < 0.05). *Orius majusculus* showed a greater preference for female adults compared to males following 24 h (*p* < 0.001) (Figure 2).

### 3.2. Atheta coriaria

**Figure 3:**
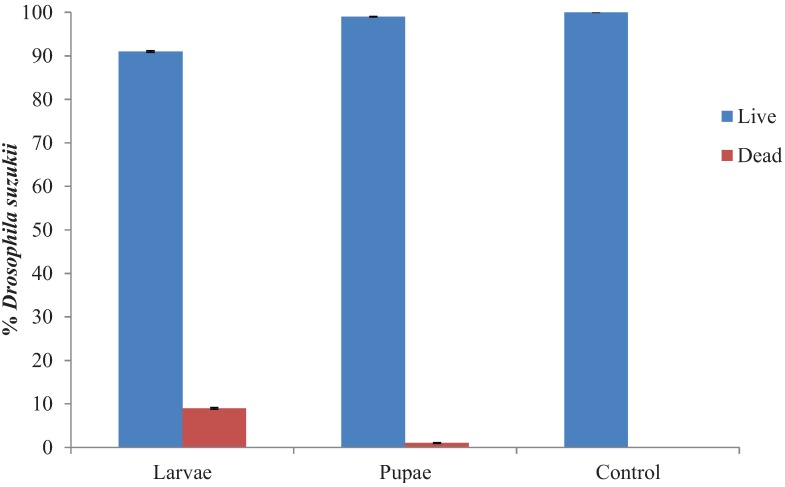
Impact of *Atheta coriaria* on various life stages of *Drosophila suzukii* following 48 h.

**Figure 4:**
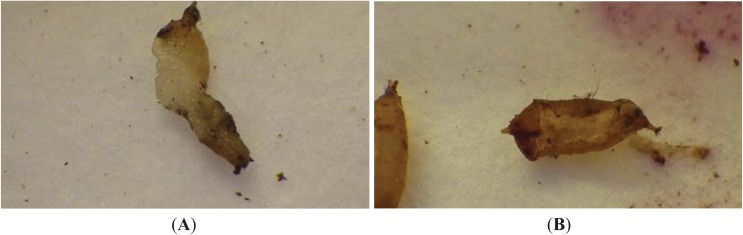
Impact of *Atheta coriaria* feeding on (**A**) *Drosophila suzukii* larvae and (**B**) a pupae (photos: Andrew G. S. Cuthbertson^©^).

*Atheta coriaria* did not have any major impact on either larvae or pupae of SWD. Only 9% of larvae were classed as having been attacked/fed upon following 48 h (Figure 3). Some pupae were observed to have been fed upon (Figure 4), but numbers were very low.

### 3.3. Hypoaspis miles

*Hypoaspis miles* had no impact upon SWD larvae or pupae. There was no sign of any feeding damage on either larvae or pupae following a period of six days. Adult flies were freely emerging from treatment (mite present) arenas (data not shown [18]).

### 3.4. Anthocoris nemoralis

**Figure 5:**
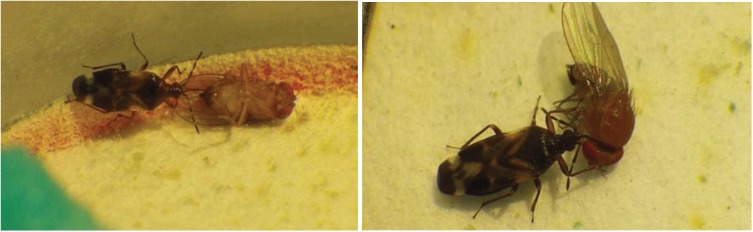
*Anthocoris nemoralis* feeding on adult *Drosophila suzukii* (photos: Andrew G. S. Cuthbertson^©^).

**Figure 6:**
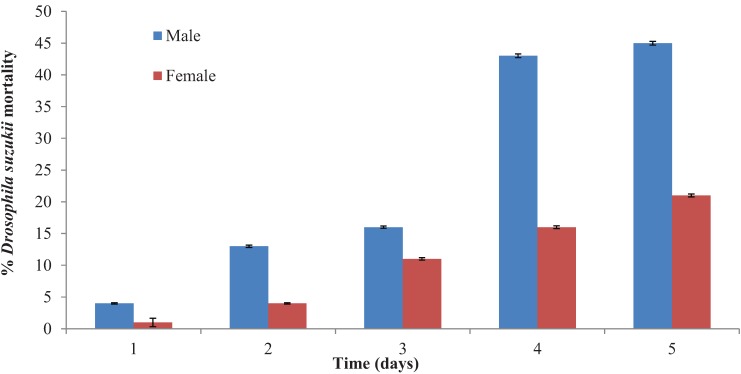
Percentage mortality of adult *Drosophila suzukii* following exposure to *Anthocoris nemoralis.*

*Anthocoris nemoralis* was also visually observed to feed on adult SWD [17] (Figure 5). Significantly more male flies were preyed upon than females (*p* < 0.001) following 24 h (Figure 6). Predation significantly (*p* < 0.001) increased against male SWD after 4 days and then appeared to level off. There was no control mortality in the female trials. The male controls suffered mortality of 36% following 5 days.

This study has evaluated several commercially available invertebrate natural enemies for their potential to act as biocontrol agents for *D. suzukii*. Out of the species screened only *A. nemoralis* and *O. laevigatus* show any potential for use in augmentative releases. The predatory mite, *H. miles* had no impact on *D. suzukii* populations.

Under laboratory conditions, within confined arenas, *A. nemoralis* shows potential for offering a measure of control on *D. suzukii* populations. Here, after 5 days 45% mortality of male SWD was obtained. This predator now requires testing under more realistic field conditions in order to prove that it can actually catch *D. suzukii* adults in the open field and not just because it was in a confined arena. This was not possible in the current study; simply due to lack of SWD numbers in the open field in the UK. It is highly likely that predatory efficiency will decrease in the open field situation due to increased difficulty in catching adult SWD and also due to the presence of other, perhaps more favourable, prey. *Orius laevigatus* also holds some potential as a control agent for *D. suzukii*. In the current study adult flies were readily attacked and fed upon (Figure 1) within experimental arenas. This, however, is in contrast to the preliminary study by Malagnini *et al*. [11] who found *O. laevigatus* to have no predatory activity against *D. suzukii*. Unpublished data cited within Walsh *et al*. [2] states that *O. insidiosus* was visually observed in the laboratory to feed on *D. suzukii* larvae infesting blueberries. Therefore, *Orius* spp., as shown in the current study, may have potential as control agents in helping to suppress *D. suzukii* populations.

*Atheta coriaria* did prey upon the larvae and pupae of *D. suzukii*, proving that should they encounter such life stages falling from soft fruit trees they can and will feed upon them. However, it would appear that they would not significantly impact populations.

Even though several of the species of natural enemies screened fed upon various life stages of *D. suzukii*, none would appear to offer sufficient control of the pest. However, as they naturally occur within the ecosystem in which *D. suzukii* occupies they are all likely to contribute to population suppression in some form. Therefore, conserving of all beneficial species populations within a given ecosystem will be of utmost importance in aiming to suppress *D. suzukii* populations.

## 4. Conclusions

*Drosophila suzukii* remains a serious threat to the UK soft fruit industry. All potential control/eradication methods and components must be fully evaluated. None of the individual species of natural enemy evaluated in the current study offered control of *D. suzukii*. However, in the open field they will all play a role in helping to suppress populations, therefore, the conservation of their populations must be actively encouraged.
